# Generation of light-producing somatic-transgenic mice using adeno-associated virus vectors

**DOI:** 10.1038/s41598-020-59075-3

**Published:** 2020-02-07

**Authors:** Rajvinder Karda, Ahad A. Rahim, Andrew M. S. Wong, Natalie Suff, Juan Antinao Diaz, Dany P. Perocheau, Maha Tijani, Joanne Ng, Julien Baruteau, Nuria Palomar Martin, Michael Hughes, Juliette M. K. M. Delhove, John R. Counsell, Jonathan D. Cooper, Els Henckaerts, Tristan R. Mckay, Suzanne M. K. Buckley, Simon N. Waddington

**Affiliations:** 1 Gene Transfer Technology Group, Institute for Women’s Health, University College London, London, UK; 2 UCL School of Pharmacy, University College London, London, UK; 3 Institute of Psychiatry, Psychology & Neuroscience, King’s College London, London, UK; 4 Department of Infectious Diseases, School of Immunology and Microbial Sciences, King’s College London, London, UK; 5 Robinson Research Institute, University of Adelaide, Adelaide, Australia; 6 Dubowitz Neuromuscular Centre, Molecular Neurosciences Section, Developmental Neurosciences Programme, UCL Great Ormond Street Institute of Child Health, London, UK; 7 NIHR Great Ormond Street Hospital Biomedical Research Centre, London, UK; 8 Department of Pediatrics, Washington University in St Louis, St Louis, MO USA; 9 Laboratory of Viral Cell Signalling and Therapeutics, Department of Cellular and Molecular Medicine and Department of Microbiology, Immunology and Transplantation, KU Leuven, 3000 Leuven Belgium; 10 Centre for Biomedicine, Manchester Metropolitan University, Manchester, UK; 11 Wits/SAMRC Antiviral Gene Therapy Research Unit, Faculty of Health Sciences, University of the Witwatersrand, Johannesburg, South Africa

**Keywords:** Gene expression, Gene regulation

## Abstract

We have previously designed a library of lentiviral vectors to generate somatic-transgenic rodents to monitor signalling pathways in diseased organs using whole-body bioluminescence imaging, in conscious, freely moving rodents. We have now expanded this technology to adeno-associated viral vectors. We first explored bio-distribution by assessing GFP expression after neonatal intravenous delivery of AAV8. We observed widespread gene expression in, central and peripheral nervous system, liver, kidney and skeletal muscle. Next, we selected a constitutive SFFV promoter and NFκB binding sequence for bioluminescence and biosensor evaluation. An intravenous injection of AAV8 containing firefly luciferase and eGFP under transcriptional control of either element resulted in strong and persistent widespread luciferase expression. A single dose of LPS-induced a 10-fold increase in luciferase expression in AAV8-NFκB mice and immunohistochemistry revealed GFP expression in cells of astrocytic and neuronal morphology. Importantly, whole-body bioluminescence persisted up to 240 days. We have validated a novel biosensor technology in an AAV system by using an NFκB response element and revealed its potential to monitor signalling pathway in a non-invasive manner in a model of LPS-induced inflammation. This technology complements existing germline-transgenic models and may be applicable to other rodent disease models.

## Introduction

Germline light-producing transgenic mice where luciferase expression is controlled by an endogenous promoter, a surrogate promoter or by a minimal promoter downstream of tandem, synthetic, transcription factor binding elements, are used to provide an *in vivo* readout of physiological and pathological processes^1,2^. One of the advantages of this technology is that every cell will contain a copy of the luciferase transgene and therefore provide a whole-body transgene expression profile under the control of a specific promoter of choice. However, producing germline transgenics requires frequent backcrossing and therefore becomes a time-consuming and costly process, using many rodents.

We have previously developed a novel technology which allows the generation of light-producing somatic transgenic rodents, using lentiviral vectors as a proof-of-concept system^3^ and have validated this technology both *in vitro*^4,5^ and *in vivo*^1,6^. We have also demonstrated that signalling pathways in diseased organs can be monitored specifically, continually and in a non-invasive manner^1,2^. Exploiting the immune tolerance of neonatal mice towards transgenic proteins^7^, we were able to achieve organ-specific transduction by a single neonatal administration of the biosensor.

To achieve a better spread of delivery and target additional tissues, we explored adeno-associated viral (AAV) vectors to deliver the biosensor construct. Previous work has shown that AAV8 can achieve widespread transgene expression and cross the blood brain barrier (BBB) after an intravenous administration in adult rodents, targeting the brain, heart, liver and skeletal muscles^8^. Neonatal intraperitoneal administration of an AAV8 vector resulted in expression in pancreas^9^, kidney^10^ and spinal cord^11^.

In this study, we have shown extensive, systemic gene expression after a single neonatal intravenous administration of AAV8-CMV-eGFP to neonatal mice. We have generated AAV8 biosensors carrying either an NFκB response element or a constitutive SFFV promoter driving luciferase. Here for the first time we report the generation of light-emitting somatic transgenic rodents with a wider spread of transgene expression, following a single neonatal intravenous or intracranial administration of AAV8 biosensors. We validated the biosensing technology by administering LPS to model systemic inflammation and showed a significant increase in light emission. This technology will allow for expedited investigations regarding signalling pathways activated in disease processes and complement existing germline transgenic light-producing technology by maximising the use, and reducing the numbers of animals used in biomedical research.

## Results

### Neonatal intravenous administration of AAV8-CMV-GFP vector

We sought to determine the bio-distribution of GFP expression following neonatal (Post-neonatal day 1, P1) intravenous administration of AAV8-CMV-eGFP. On the day of birth, mice received 40μl of vector (1 × 10^13^ vector genomes/ml) via the superficial temporal vein. One month later, mice were harvested and GFP expression was analysed, using a DFC420 microscope.

**Figure 1:**
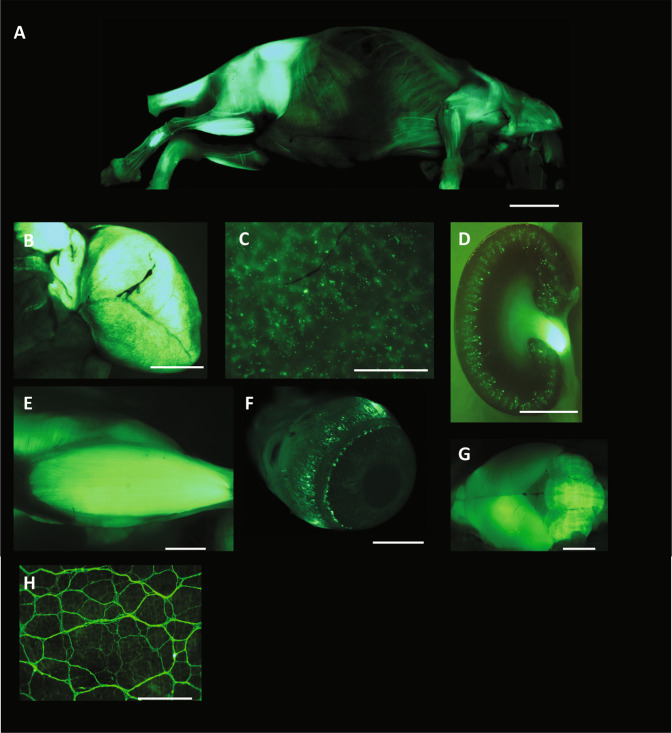
Systemic distribution of GFP after a single neonatal intravenous administration of AAV8-CMV-GFP. New-born mice received an intravenous administration of AAV8-CMV-GFP vector (n = 3). At 1 month of age the mice were harvested and the *ex vivo* analysis of GFP expression revealed widespread systemic distribution. (**A**) Strong GFP expression was observed within the heart (**B**), liver (**C**), kidney (**D**), muscle (**E**), eye ball (**F**), brain (**G**) and the myenteric plexus. (**H**) Scale bar = 1.80 µm for A. Scale bar = 2.5 µm for B, D, E, F and G. Scale bar = 3 µm for C and H.

Prior to gross dissection and further fixation, skin was removed and mice were viewed under a stereoscopic fluorescence microscope. Strong, extensive and widespread GFP expression was observed throughout the body, although the highest levels of expression were most noticeable in the musculature (Fig. 1). Strong transduction was observed within the heart (B), liver (C), kidney (D), skeletal muscle (E), eye (F), brain (G), and myenteric plexus (H). At higher magnification, we observed a majority of GFP positive hepatocyte cells in the liver (Supplementary Fig. 1).

In order to assess the expression profile in the CNS, the brains from injected and non-injected control mice were sectioned and immunohistochemistry was conducted for GFP expression. This revealed extensive and widespread GFP expression (Supplementary Fig. 2). Further examination under higher magnification of discrete areas of the brain including the primary motor cortex, the somatosensory barrel field (S1BF), piriform cortex, dentate gyrus, cerebellum, and the gigantocellular nucleus revealed transduction of cells with both neuronal and glial morphology (Supplementary Fig. 2).

Further to this we investigated whether AAV vector or GFP transgene expression triggers an inflammatory response after neonatal intracranial injections. Microglial activation was examined in all brains collected at 35 days of development and compared to brain tissue from Ppt1^−/−^ (palmitoyl protein thioesterase 1) mice in which profound microglial activation occurs^12^, and therefore serve as a positive control for microglial markers. Extensive microglial engorgement and activation was observed in the Ppt1^−/−^ mice, and no noticeable activation of microglia was observed in the non-injected and AAV8 injected brains (Supplementary Fig. 3).

### Production of AAV8 biosensors

An AAV8 producer plasmid was created containing a Gateway® accepter site (Invitrogen). The Gateway® sequence was cloned into the backbone and was placed upstream of a minimal promoter driving a codon-optimised luciferase transgene and an enhanced GFP linked by a bicistronic linker, T2A (Supplementary Fig. 4). We have now assembled an extensive library of transcription factor binding elements in pENTR shuttle plasmids and these are shown in Supplementary Fig. 4. We selected the NFκB response element and an SFFV viral promoter for the insertion into the AAV gateway backbone. These two were chosen as they have been validated by both *in vitro* and *in vivo* means in our lentiviral system^3^.

AAV8 biosensor vectors were generated using the AAV8-SFFV-Luc-T2A-eGFP and AAV8- NFκB -Luc-T2A-eGFP backbones.

### Neonatal administration of AAV8 biosensors

Having observed widespread transgene expression after a single neonatal administration of an AAV8-CMV-GFP vector, we chose to investigate the NFκB signalling expression profile by neonatal injection of the AAV8-NFκB-Luc-2A-GFP biosensor. We selected AAV8-SFFV-Luc-2A-GFP as a constitutively expressed control and to allow comparison with previous experiments using lentivirus vectors^3^.

At P1 of development, mice received a 30 µl intravenous (IV) administration of AAV8 SFFV or AAV8 NFκB biosensor (1 × 10^13^ vg/ml). Mice underwent whole-body bioluminescence imaging over the course of development to quantify luciferase expression.

**Figure 2:**
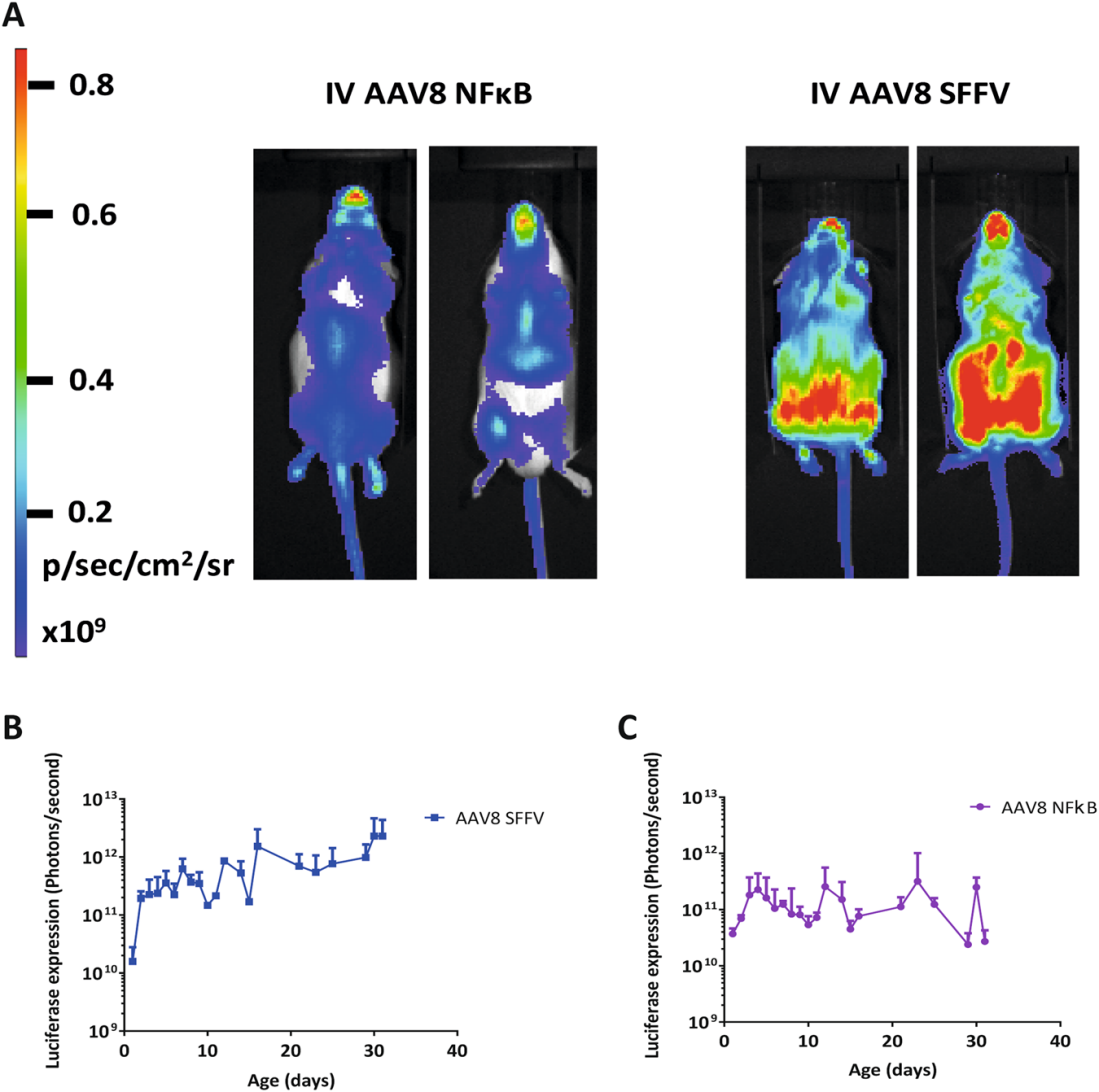
Whole-body bioluminescence imaging of mice which have received an intravenous administration of AAV8 SFFV or AAV8 NFκB vectors. New-born mice received intravenous injections of either AAV8 SFFV or AAV8 NFκB biosensors (n = 6 per group). We observed expression profiles from the two different AAV8 biosensors (**A**), the same mouse was imaged on its front and back. Luciferase expression was quantified by whole-body bioluminescence imaging for a month (**B**,**C**), (mean +/− SD).

Following IV injection of the AAV8 NFκB biosensor, luciferase expression was strongest in the spine, thorax, paws, lower abdominal and the mouth (Fig. 2A). In contrast, IV injection of the AAV8 SFFV biosensor resulted in whole-body luciferase expression but with strongest expression in the lower abdomen (Fig. 2A). The luciferase expression was quantified and showed stable transgene expression over development (Fig. 2B,C).

Additionally, to restrict the expression profile within the CNS and PNS, intracranial (IC) injections were also performed with AAV8 SFFV and AAV8 NFκB biosensors. P1 pups received 5 µl (1 × 10^13^ vg/ml) of either biosensor, and whole-body bioluminescence imaging was undertaken over the course of development. Luciferase expression from the IC injected AAV8 NFκB biosensors was similar to that seen in the IV injected mice, predominantly in the spine, thorax, paws, mouth, eyes and tail (Supplementary Fig. 6A).

A month after IC or IV injection of AAV8 SFFV or NFκB biosensor, the mice were harvested, immunohistochemistry and immunofluorescence with neuronal and astrocytic markers were performed to detect GFP expression in the brain. After IC injection of AAV8 SFFV, GFP expression was predominantly neuronal within the dentate gyrus and CA1 and CA3 regions of the hippocampus (Supplementary Figs. 7A and 8A), with some astrocytic cell targeting (Supplementary Fig. 8B). Both neuronal and astrocytic cells were transduced by IC AAV8 NFκB and IV AAV8 SFFV (Supplementary Fig. 8A,B). However, following IV AAV8 SFFV, a mixture of cells were found to be positive for GFP within the hippocampal region after immunoperoxidase staining (Supplementary Fig. 7B). After co-staining these sections with neuronal and astrocytic markers, we found no co-localisation with GFP (Supplementary Fig. 8A,B).

**Figure 3:**
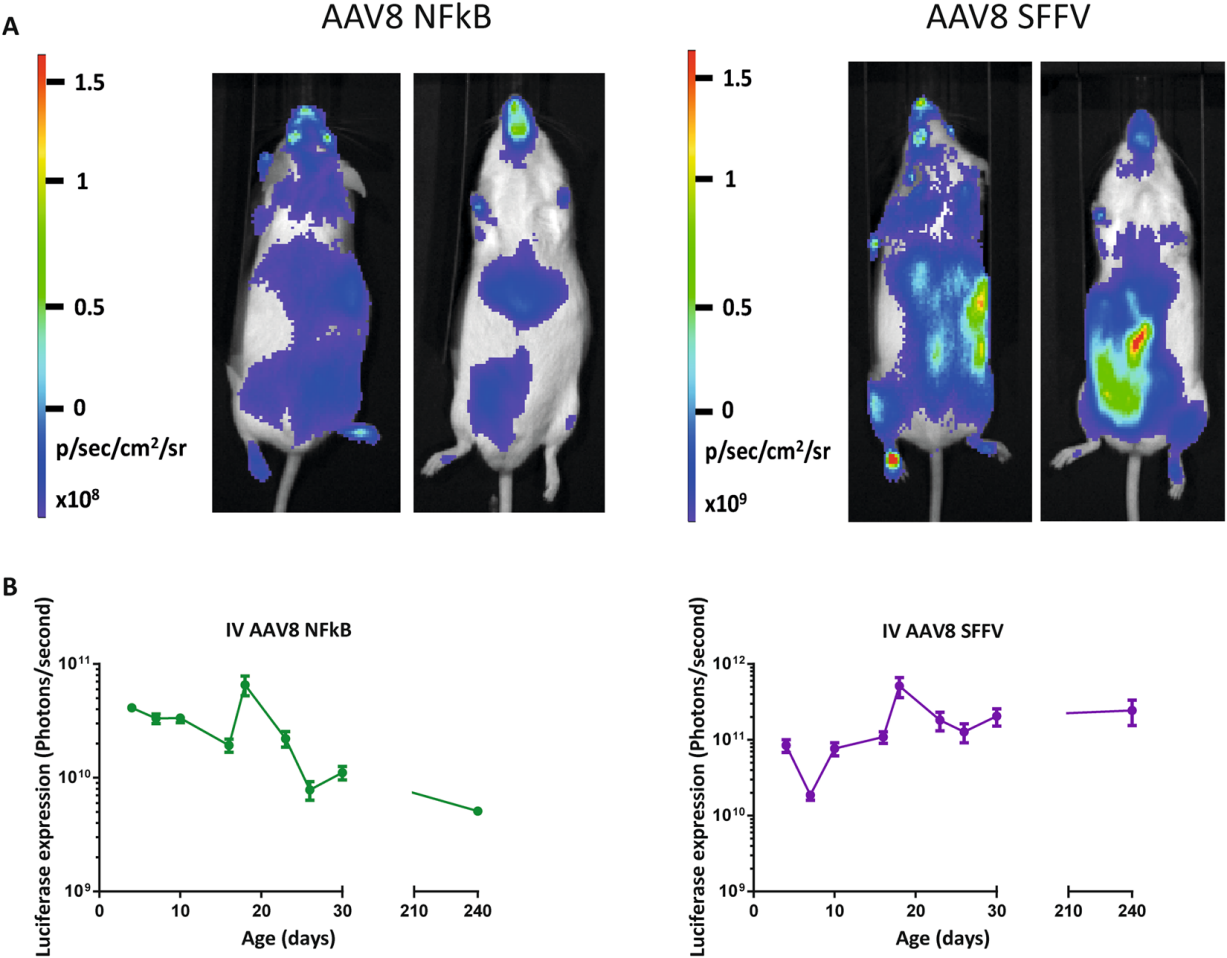
Whole-body bioluminescence imaging of mice which have received an intravenous administration of AAV8 NFκB or AAV8 SFFV vectors up to 240 days of development. New born mice received intravenous injections of AAV8 NFκB or AAV8 SFFV preformed biosensors (n = 8 per group). (**A**) the same mouse was imaged on its front and back. Luciferase expression was quantified by whole-body bioluminescence imaging for 8 months (**B**), (mean +/− SD).

Further to this, on a separate cohort of intravenously injected mice, we were able to show extensive whole-body bioluminescence at 240 days of development (Fig. 3).

### Validation of the AAV8 NFκB biosensor

**Figure 4:**
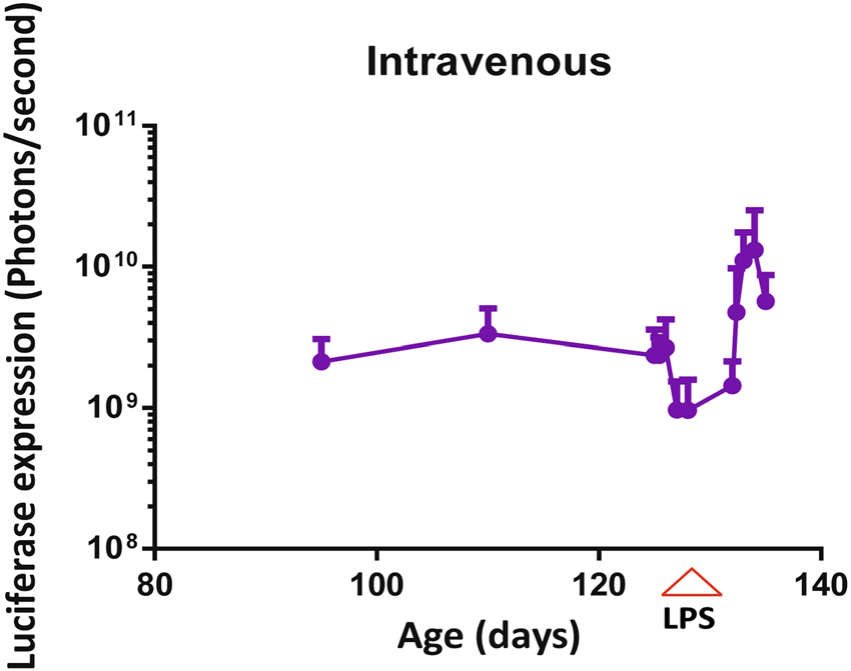
Up-regulation of luciferase signal mediated by a single dose of LPS. Bioluminescence imaging persisted more than 4 months during development in mice which received intravenous administration of the AAV8 NFκB biosensor (n = 7). At day 132 of development all the mice received a single dose of LPS which resulted in a significant up-regulation in luciferase expression in the IV injected mice, p < 0.0001.

To assess whether the AAV8 NFκB could be exploited to report on NFκB signalling in pathological states, mice received ultra-pure lipopolysaccharide (LPS) which only acts through toll-like receptor 4 to induce translocation of NFκB from the cytosol to the nucleus^13^. At day 132 after AAV administration, a single dose of LPS was administered intraperitoneally to both IV and IC injected groups. Bioimaging was taken at 4, 24, 48, 72 and 96 hours before (to correct for any perturbance in the signal caused by multiple imaging) and after LPS administration. A single administration of LPS resulted in a significant increase in luciferase expression in IV (Fig. 4; p < 0.0001, n = 7) and IC AAV8 NFκB mice (Supplementary Fig. 9; p = 0.006, n = 7). The luciferase expression for individual mice which received IV injections is shown in Supplementary Fig. 10.

## Discussion

In this study, we have shown for the first time that light-producing somatic-transgenic rodents can be produced by a single neonatal administration of an AAV8 biosensor. Specifically, we have demonstrated that AAV8 vectors cross the blood brain barrier (BBB) and mediate systemic transgene expression after a single neonatal intravenous or intracranial administration.

Our results showed a systemic spread of transgene expression by a neonatal intravenous administration of a self-complementary AAV8 vector. This data agrees with previous work conducted by Nakai *et al*. which demonstrated that AAV8 vectors cross the BBB and result in transduction of both neuronal and glial cells in the brain, albeit with a low efficiency^14^. These authors also presented transduction of cells within the liver, skeletal muscle, smooth muscle, cardiac muscle and pancreas^14^. However, we were able to show a much more extensive spread of transgene expression within the CNS and visceral organs such as the kidney and myenteric plexus. The strong and widespread GFP expression observed in our study maybe due to a number of factors including the use of a different promoter and route of administration.

Our analysis also demonstrated an improved safety profile within the CNS after a neonatal intravenous administration. It has been reported that lentiviral vector mediated expression of GFP elicits toxicity in Purkinje cells of the developing mouse cerebellum. This toxicity was attributed mainly to GFP overexpression although the authors could not discount the lentiviral vectors *per se*, or genotoxicity caused by vector integration in neuronal DNA as aggregating factors^15^. In contrast, we detected no microglial activation in the CNS suggesting that inflammation or gliosis caused by the injection, the presence of vector, or expression of both luciferase and GFP did not occur. This suggests that the AAV system we describe may be preferable to our previously described lentiviral vector system^2^.

We performed neonatal intracranial injections of our AAV8 biosensors to monitor whether we could restrict the expression profile to the CNS and PNS. However, our results revealed that the intracranial injected mice showed a similar expression profile to the mice which received an intravenous administration. Previous work has shown that after a single adult intravenous injections of AAV8, transgene expression was observed within the CNS and also within the cervical, thoracic, and lumbar spinal cord sections. However, the expression profile was not significant compared to other serotypes such as AAVrh.8^16^.

In order to overcome this wide distribution profile, future experiments would involve the assessment of additional AAV capsids. These would include AAV-PHP.B, which is a new variant AAV capsid that transduces the CNS 40-fold greater than AAV9 after a single adult intravenous administration and targets astrocytes and neurons^17^. Furthermore, AAV-PHP.eB an enhanced variant of AAV-PHP.B, efficiently transduces the CNS and PNS greater than the AAV-PHP.B and predominately targets neurons^18^. Adult injections would further restrict the transgene expression in the CNS. Kostoula *et al*. demonstrated CNS specific luciferase expression after adult stereotactic injections of AAV8-GFAP-luciferase into the hippocampus^19^. Therefore, in order to achieve CNS restricted and cell specific transduction profiles different configurations of AAV capsids and adult injections would have to be used.

We have established a unique technology which allows the production of somatic-transgenic light-emitting rodents. We have synthesised a library of >20 response elements, >10 promoters, most of which we have validated in the context of a lentiviral backbone *in vitro*, and several of them *in vivo*. To exploit the advantages of AAV, we gene synthesised an AAV backbone containing a Gateway acceptor site, and strategically placed multi-cloning sites with unique restriction sites to permit easy cloning.

As proof-of principle we inserted the NFκB response element, consisting of octuplet cognate binding elements, into the AAV backbone. A potential concern with introducing such sequences into the viral backbone is the loss of tandem repeats following propagation and amplification in bacteria^20^. However, sequencing of large-scale DNA preparations of this backbone confirmed that the repeats remained intact.

Here we demonstrate that administration of AAV biosensors by a single neonatal intravenous administration resulted in widespread luciferase expression, in comparison to our lentiviral biosensors which only transduced the liver^3^. The bio-distribution of luciferase expression differed between the constitutive AAV8 SFFV biosensor and the NFκB biosensor. A previous study demonstrated NFκB biosensor activity following administration of AAV by pancreatic infusion in adult mice^21^. Baseline expression rose rapidly in those mice but fell to modest levels by 1 month. This may be attributable to an anti-luciferase or anti-capsid cytotoxic T-cell response eliminating transduced cells. In contrast, we observed stable expression for at least five months; it has been shown that neonatal gene transfer administration results in immune tolerization to the transgenic protein when using retrovirus^22^, AAV^23^ and adenovirus^24^ vectors.

We also conducted additional studies to test another AAV8 biosensor *in vivo*. We administered an AAV8 biosensor containing a hypoxic response element (HRE), driving luciferase via tail vein injections to adult mice (8 weeks). At day 77 post injection, the mice were subjected to brief hypoxia (10% oxygen)^25^. A burst of bioluminescence imaging was taken pre- and post-hypoxia and the results revealed no difference in luciferase expression after hypoxia (Supplementary Fig. 11). Therefore, highlighting that certain AAV biosensors aren’t as sensitive as germline transgenic mice in detecting subtle changes in cellular signalling.

Here we present for the first time the generation of somatic-transgenic mice with the use of AAV viral vectors delivered to neonatal mice. We have also shown widespread transgene expression after a single intracranial or intravenous neonatal administration of the AAV biosensors. We have validated the AAV biosensors with the use of agonists. This technology not only complements existing germline transgenic rodents but also maximises the use and reduces the numbers of animals used in biomedical research^26^. We have generated a non-invasive gene marking technology, which can be applied to systemic disease models. With the use of Gateway® cloning the assessment of different response elements can easily be achieved.

## Methods and Materials

All methods were performed in accordance with the relevant guidelines and regulations.

### Generation of the AAV biosensor plasmids

The AAV8-Gateway®-Luc-T2A-eGFP was synthesised by Aldevron (Aldevron, North Dakota, USA). This AAV plasmid consisted of a Gateway® sequence, placed upstream of a codon-optimised luciferase transgene, linked to an enhanced GFP by a bicistronic linker, T2A. The response element NFκB and constitutive promoter SFFV was cloned into an pENT plasmid^3^. By using the Gateway® cloning kit (Invitrogen, Manchester, UK) and using the manufactures guidelines, the response element NFκB, HRE and constitutive promoter SFFV were individually inserted into the AAV backbone to generate the following plasmids; pAAV8-SFFV-Luc-T2A-eGFP, pAAV8-NFκB-Luc-T2A-eGFP and pAAV8-HRE-Luc-T2A-eGFP.

### Recombinant AAV production

Recombinant AAV was produced, purified and titered using standard procedures. Briefly, HEK293T cells were double transfected with the pAAV8-SFFV-Luc-2A-eGFP or pAAV8-NFκB -Luc-2A-eGFP or pAAV8-HRE-Luc-T2A-eGFP plasmid, and the pDG8 plasmid expressing AAV2 Rep, AAV8 Cap gene and adenovirus 5 helper functions (Plasmid Factory, Bielefeld, Germany) using polyethylenimine (PEImax, Polysciences Inc). After 72 hours of incubation at 37 °C, the cells were harvested by centrifugation and then lysed by freeze-thawing in lysis buffer. In parallel, the virus-containing supernatant was harvested and precipitated by using ammonium sulphate salt. Cell lysate and precipitated supernatant were treated with benzonase, clarified by centrifugation and filtered at 0.22 µm before purification.

The recombinant AAV virus preparations were purified by iodixanol step gradient: the viral preparation was overlaid with increasing concentrations of iodixanol (15%, 25%, 40% and 60%, OptiPrep; Sigma-Aldrich, Dorset, UK). The tubes were centrifuged at 40,000 rpm for 3 hours at 18 °C in a Sorvall Discovery 90SE ultracentrifuge using a TH641 rotor (Thermo Scientific, Paisley, UK). The vector was extracted from the 40% fraction with a 19-gauge needle. Purified vector fractions were dialysed against PBS overnight.

Real-time PCR and alkaline gel electrophoresis were used to assess the viral genome titers and integrity of the viral genome^27,28^, the capsid titers were determined by SDS PAGE electrophoresis^29^.

AAV8 containing the enhanced GFP gene driven by the cytomegalovirus promoter (AAV8-CMV-eGFP) in a self-complementary configuration was obtained from the UPenn Vector Core facility (Philadelphia, USA) at a titre of 1 × 10^13^ vg/ml.

### Animal procedures

Outbred CD1 mice and MF1 mice used in this study were supplied by Charles Rivers Laboratories. All procedures were performed under United Kingdom Home Office Project License 70/8030, approved by the ethical review committee and followed institutional guidelines at University College London. All methods were performed in accordance with the relevant guidelines and regulations.

### Neonatal Intracranial and Intravenous injections

For intracranial injections, mice (random mix of males and females) were subjected to brief hypothermic anaesthesia on the day of birth, followed by unilateral injections of concentrated AAV vector (5 µl in total; 1 × 10^13^ vector genomes/ml) into the cerebral lateral ventricles using a 33 gauge Hamilton needle (Fisher Scientific, Loughborough, UK), following co-ordinates provided by Kim *et al*.^30^. For intravenous injections, pups were subjected to brief hypothermic anaesthesia followed by intravenous injections of AAV vectors into the superficial temporal vein^31^, with a total volume of 25 µl (1 × 10^13^ vg/ml). The neonates were then allowed to return to normal temperature before placing them back with the dam.

### Adult tail vein injections

Adult male CD1 mice were placed in an chamber set at 37 °C, after which they were anaesthetised with isoflurane with 21% oxygen (Abbotts Laboratories, London, UK). A total volume of 100 µl (1 × 10^10^ vg/ml) of AAV8-HRE-Luc-T2A-eGFP was administered via tail vein to each mouse. The mice were then placed back into their cages.

### Whole-body bioluminescence imaging

Where appropriate, mice were anaesthetised with isoflurane with 21% oxygen. D-luciferin (Gold Biotechnologies, ST Louis, USA) was administered by intraperitoneal injection at a concentration of 15 mg/mL. Mice were imaged 5 minutes after luciferin injection using a cooled charged-coupled device camera, (IVIS Lumina II, Perkin Elmer, Coventry, UK) for between 1 second and 5 minutes. Photon output of the whole-body was measured using Living Image Software (Perkin Elmer) and light output quantified and expressed as photons per second per centimetre squared per steradian (photons/second/cm^2^/sr).

### Hypoxia

Adult mice (19 week old mice) were placed inside a hypoxic chamber which was air tight. The mice were subjected to 10% oxygen mixed with nitrogen for 2 hours following protocols mentioned in Karda *et al*.^2^ and Nakada *et al*.^25^. The mice were then returned to their dams.

### Collection of brain tissues

Mice were anaesthetised at day 35 of development using Isoflurane and the right cardiac atrium was incised, followed by injection of heparinized PBS into the left cardiac ventricle. The brains were removed and fixed in 4% paraformaldehyde (PFA) and then cryoprotected in 30% sucrose in 50 mM PBS. Brains were sectioned using a sliding microtome (Carl Zeiss, Welwyn Garden City, UK) to generate 40 µm transverse sections^31^. Sections were stored in 30% sucrose in TBS, ethylene glycol and 10% sodium azide.

### Immunoperoxidase immunohistochemistry

To visualise CD68 and GFP immunoreactivity, sections were treated with 30% H_2_O_2_ in TBS for 30 minutes. They were blocked with 15% rabbit serum for CD68 (Vector Laboratories, Cambridge, UK) and goat serum for GFP (Vector Laboratories) in Tris buffered saline and Tween 20 (TBST) for 30 minutes. This was followed by the addition of primary antibody, rat anti-mouse CD68 (1:100; Biorad, Hertfordshire, UK), mouse anti-GFP (1:10,000; Abcam, Cambridge, UK) in 10% serum and TBST and incubated over night at 4 °C with constant gentle aggitation. The following day the sections were treated with the secondary rat anti-rabbit antibody for CD68 (1:1000; Vector Laboratories) and goat anti-rabbit (1:1000 dilution; Vector Laboratories) in 10% rat or goat serum in TBST for 2 hours. The sections were incubated for a further 2 hours with Vectastain ABC (Vector Laboratories). 0.05% of 3,3′-diaminobenzidine (DAB) was added and left for a couple of minutes. Sections were transferred to ice cold TBS. Individual brain sections were mounted onto chrome gelatine-coated Superfrost-plus slides (VWR, Poole, UK) and left to dry for 24 hours. The slides were dehydrated in 100% ethanol and placed in Histoclear (National Diagnostics, Yorkshire, UK) for 5 minutes before adding a coverslip with DPX mounting medium (VWR). DAB stained sections in Supplementary Figs. 1 and 2 were viewed using an Axioskop 2 Mot microscope (Carl Zeiss Ltd.) and representative images were captured using an Axiocam HR camera and Axiovision 4.2 software (Carl Zeiss Ltd.). DAB stained sections in Supplementary Fig. 7 were viewed using Leica MZ16F microscope software.

### Fluorescence immunohistochemistry

A similar protocol was followed as with the immunoperoxidase stain; however H_2_O_2_ treatment was omitted. Followed protocol mentioned in Karda *et al*.^2^.

### Quantitative analysis of immunohistological staining

GFP and CD68 expression was quantified by thresholding analysis as previously described^12,32^. Briefly, 40 non-overlapping RGB images were taken from four consecutive sections through the somatosensory barrel field (S1BF), caudate putamen (Cpu), the *Cornu Ammonis* region 1 of the hippocampus, (CA1), piriform cortex (piriform cort), and the 10Cb region of the cerebellum (10Cb). The images were captured using a live video camera (JVC, 3CCD, KY-F55B) mounted onto a Zeiss Axioplan microscope using the x40 objective lens. All camera and microscope calibration and settings were kept constant throughout the image capture period. Images were analysed for optimal segmentation and immunoreactive profiles were determined using *Image-Pro Plus* (Media Cybernetics). Foreground immunostaining was accurately defined according to averaging of the highest and lowest immunoreactivities within the sample population for a given immunohistochemical marker (per colour/filter channel selected) and measured on a scale from 0 (100% transmitted light) to 255 (0% transmitted light) for each pixel. This threshold setting was constant to all subsequent images analyzed for the antigen used. Immunoreactive profiles were discriminated in this manner to determine the specific immunoreactive area (the mean grey value obtained by subtracting the total mean grey value from non-immunoreacted value per defined field). Macros were recorded to transfer the data to a spreadsheet for subsequent statistical analysis.

### Statistics

The data from Supplementary Figs. 2 and 3 were plotted graphically as the mean percentage area of immune-reactivity per field ± S.D or S.E.M. for each region. Two-way ANOVA with and a Sidak’s multiple comparison was performed in Fig. 4 and Supplementary Fig. 9.

## Supplementary information

Supplementary figures.
