# Supplementary material for: Generation of light-producing somatic-transgenic mice using adeno-associated virus vectors
Source: Sci Rep. 2020 Feb 7;10:2121. doi: 10.1038/s41598-020-59075-3 (PMC7005886; doi:10.1038/s41598-020-59075-3)
Supplement: Supplementary file 1 — Supplementary figures. [file 41598_2020_59075_MOESM1_ESM.pdf]

Generation of light-producing somatic-transgenic mice using adeno-associated virus vectors

Rajvinder Karda[1], Ahad A. Rahim[2], Andrew M.S. Wong[3], Natalie Suff[1], Juan Antinao Diaz[1], Dany P. Perocheau[1],Maha Tijani[1], Joanne Ng[1], Julien Baruteau[1], Nuria Palomar Martin[4], Michael Hughes[2], Juliette M.K.M. Delhove[5], John R. Counsell[6,7], Jonathan D. Cooper[3,8], Els Henckaerts[4,9], Tristan R. Mckay[10], Suzanne M.K. Buckley[1] & Simon N. Waddington[1,11]

1. Gene Transfer Technology Group, Institute for Women's Health, University College London, UK
2. UCL School of Pharmacy, University College London, UK
3. Institute of Psychiatry, Psychology & Neuroscience, King's College London, UK
4. Department of Infectious Diseases, School of Immunology and Microbial Sciences, King's College London, UK
5. Robinson Research Institute, University of Adelaide, Adelaide, Australia
6. Dubowitz Neuromuscular Centre, Molecular Neurosciences Section, Developmental Neurosciences Programme, UCL Great Ormond Street Institute of Child Health, London, UK.
7. NIHR Great Ormond Street Hospital Biomedical Research Centre, London, UK
8. Department of Pediatrics, Washington University in St Louis, St Louis, MO, USA
9. Laboratory of Viral Cell Signalling and Therapeutics, Department of Cellular and Molecular Medicine and Department of Microbiology, Immunology and Transplantation, KU Leuven, 3000 Leuven, Belgium
10. Centre for Biomedicine, Manchester Metropolitan University, Manchester, UK
11. Wits/SAMRC Antiviral Gene Therapy Research Unit, Faculty of Health Sciences, University of the Witwatersrand, Johannesburg, South Africa.

Supplementary Figure 1

A B

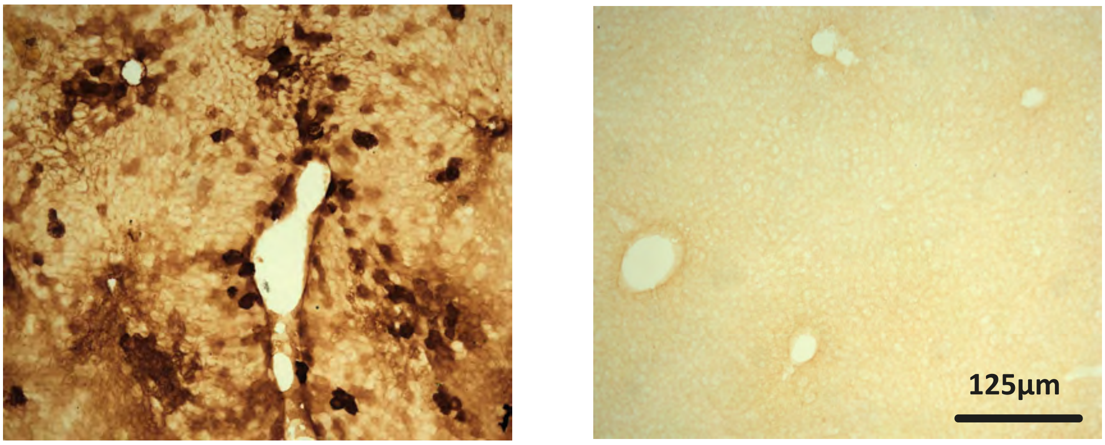

Supplementary figure 1 – GFP positive cells in the liver after neonatal intravenous delivery of AAV8-CMV-eGFP. Liver sections from injected mice were used to determine GFP positive cells. IV AAV8-CMV-eGFP revealed a predominant hepatocytes (A). There was no GFP positive cells in the un-injected control (B). Scale bar, 125µm.

Supplementary Figure 2

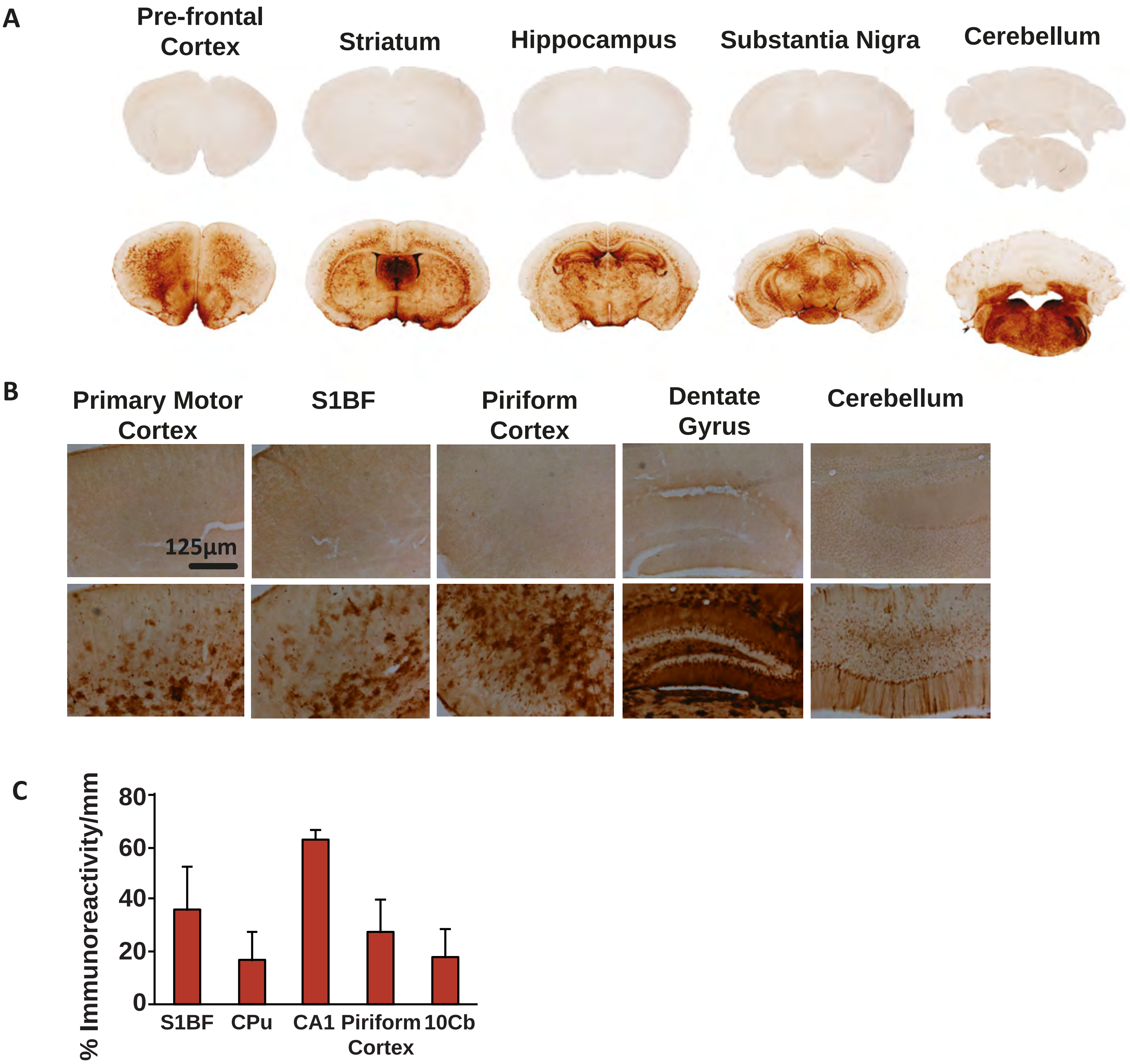

Supplementary figure 2 - Immunohistochemical detection and quantification of GFP expression 1 month post intravenous injection of AAV8 into P1 neonates. P1 neonates were intravenously administered with self-complementary AAV8–CMV-eGFP (n=3). Uninjected mice were used as negative controls (n=3). At P30 the mice were culled and the brains were removed for sectioning and immunohistochemical staining using anti-GFP antibody and DAB staining. The sections were examined by light microscopy and representative images were taken from the pre-frontal cortex, striatum, hippocampus, piriform lobes and the cerebellum (A). Various discrete areas of the brains were examined under higher magnification including the primary motor cortex, S1BF, piriform cortex, dentate gyrus, cerebellum and gigantocellular nucleus (B). Quantitative measurement of staining in discrete areas of the brain was conducted by thresholding analysis in the S1BF, caudate putamen (CPu), CA1 region of the hippocampus (CA1), piriform cortex and the cerebellar nodule (10cb). The data was plotted as the mean ± S.D. Scale bar 125µm.

Supplementary Figure 3

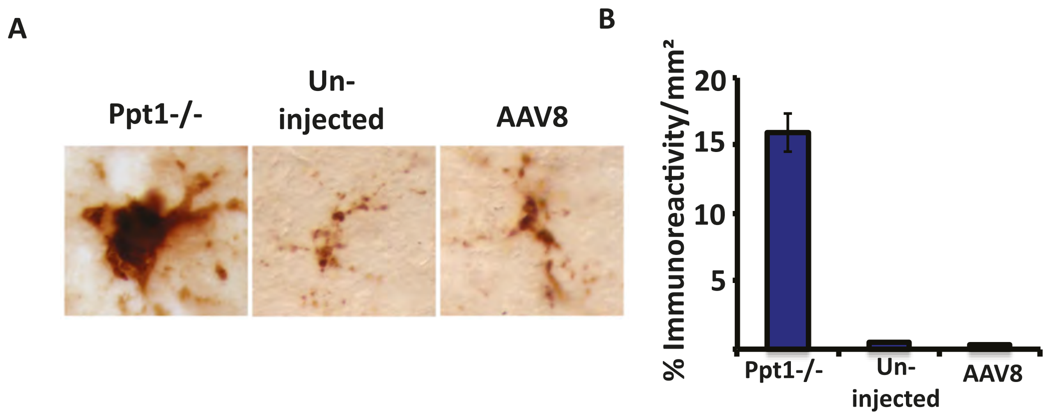

Supplementary figure 3. Immunohistochemical and quantitative analysis of microglia activation. Brain sections from both injected and uninjected mice were analysed for microglia-mediated immune response (tissue collected at P35). The sections were probed using antibodies against the microglia marker CD68 and detected using DAB. As a positive control, brain sections taken from Ppt1-/- knockout mice (n=3) were included since they have a known microglia immune response. Representative high magnification images were taken of stained microglia from Ppt1-/- mice, uninjected mice and AAV8 administered mice (A). Quantitative measurement of staining intensity was conducted by thresholding analysis (B). The data is plotted as the mean ± SEM (n=3).

Supplementary Figure 4

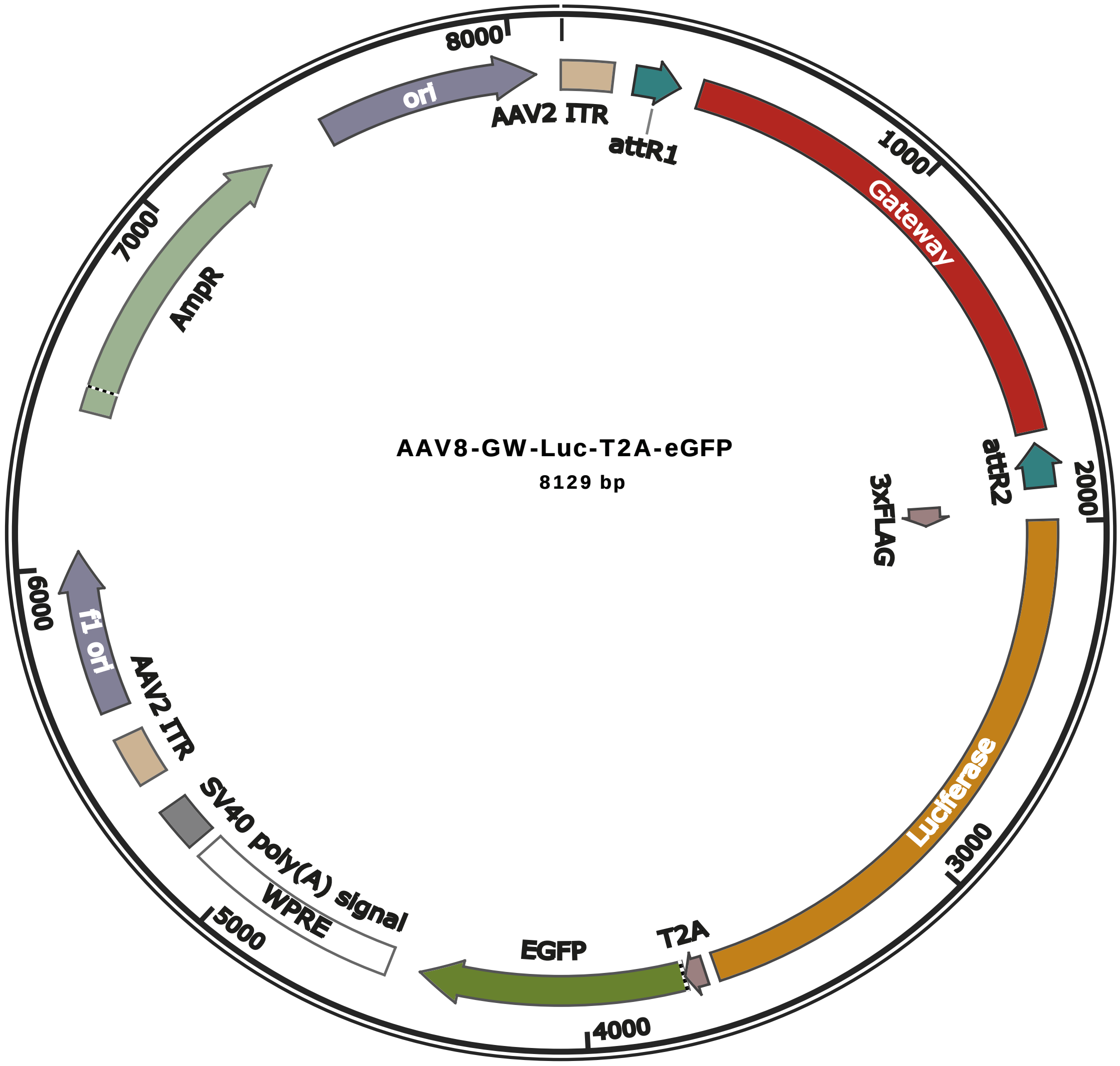

Supplementary figure 4– Plasmid configuration for AAV8-GW-Luc-T2A-GFP. Between the AAV2 ITR sequences is the Gateway cloning site placed upstream of a minimal promoter, followed by a 3xFlag tag upstream of the codon-optimised luciferase transgene. A bicistronic linker, T2A sequence, is placed upstream of an enhanced GFP. Gene expression is enhanced with the presence of woodchuck hepatitis virus post-transcriptional regulatory element (WPRE).

Supplementary Figure 5

Response Element	Disease Model	Response Element sequence	*In vitro* agonist
**NFκB**	Inflammation/Cancer	(GGGACTTTCC) x8	LPS
**SMAD 2/3 (TGF-β)**	Fibrosis	(AGCCAGACA) x8	Activin A
**SMAD 1/5/8 (BMP)**	Cancer/EMT	(CGCGGCGCCAGCCTGACAGCCCG) x6	BMP2a
**Hypoxia**	Ischemia/Cancer	(TACGTGCT) x8	Low O_2
**TCF/LEF (Wnt)**	Development	(AGATCAAAGGGGGTA) x8	$LiCl_2$
**Estrogen**	Cancer	(GTCAGGTCACAGTGACCTGAT) x4	Estradiol
**p53**	Cancer	(AGACATGTCCAGACATGTCCGAACATGTCCCAACATGTTGT) x4	Nutlin3
**AP-1**	Cancer	(TGAGTCAG) x8	PMA
**PI3K/Akt**	Cancer/Development	(GATCAAGTAAACAACTATGTAAACAA) x4	LY294002
**STAT3**	Cancer/Development	(GTCGACATTTCCCGTAAATCGTCGA) x4	IL-6
**GLI-1 (Hedgehog)**	Development	(GACCACCCAC) x8	Purmorphamine
**HNF4-β**	Development/Toxicity	(GGCAAAGGTCAT) x8	Linoleic Acid
**Antioxidant (NRF2)**	Toxicity	(TCACAGTGACTCAGCAAAATT) x8	H_2O_2
**Xenobiotic**	Toxicity	(TGAGTTCTCACGCTAGCAGAT) x8	TCDD
**NFAT**	Immunity	(GGAGGAAAAACTGTTTCATACAGAAGGCGT) x4	PMA
**Glucocorticoid**	Cardiovascular/Immunity	(GGTACATTTTGTTCT) x8	Dexamethasone
**Notch**	Cancer/Development	(CGTGGGAA) x8	NICD
**LXR**	Hepatic Regeneration	(TGAATGACCAGCAGTAACCTCAGC) x6	Omeprozole
**ISL1**	Development	(TTAATGANNNNNNNNNNCTAATGA) x4	Insulin
**TFEB**	Autophagy	(TCACGTGA) x8	Serum starvation

Supplementary figure 5– Table of response element cloned into the lentiviral biosensor. A number of response elements are cloned into the lentiviral backbone using Gateway® cloning. Each response element has been validated *in vitro* with the appropriate agonist.

Supplementary Figure 6

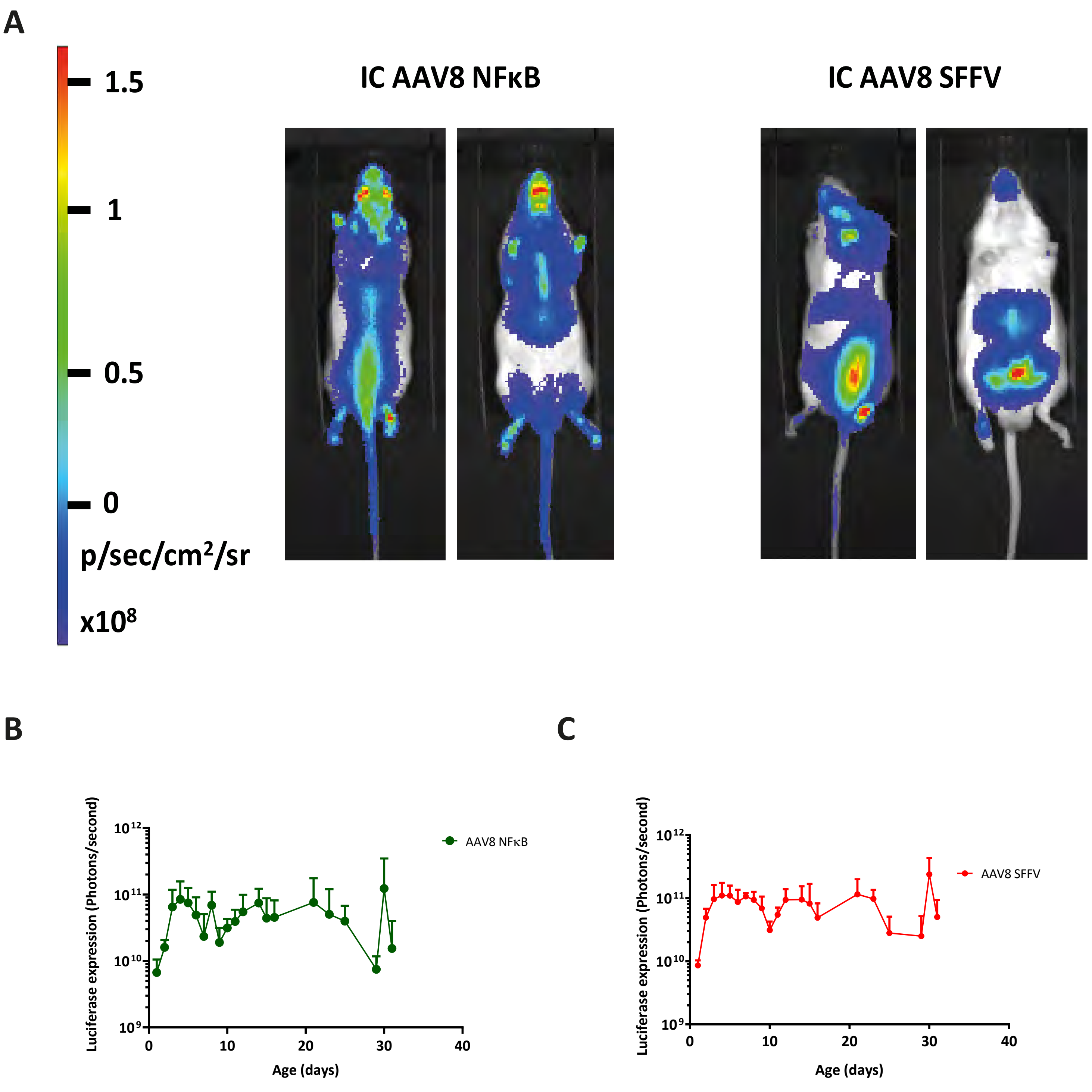

Supplementary figure 6- Luciferase expression and bio-distribution following intracranial injections of AAV8 NFκB or AAV8 SFFV vectors. Mice received an intracranial administration of AAV8 NFκB or SFFV vectors (n = 6 per group) on the day of birth, P1. The mice underwent whole-body bioluminescence imaging over development up to day 31 of development. The bio-distribution of luciferase expression from the two biosensors differed (A), the same mouse was imaged on its front and back. Luciferase expression was quantified for a month (B and C) (mean +/- SD).

Supplementary Figure 7

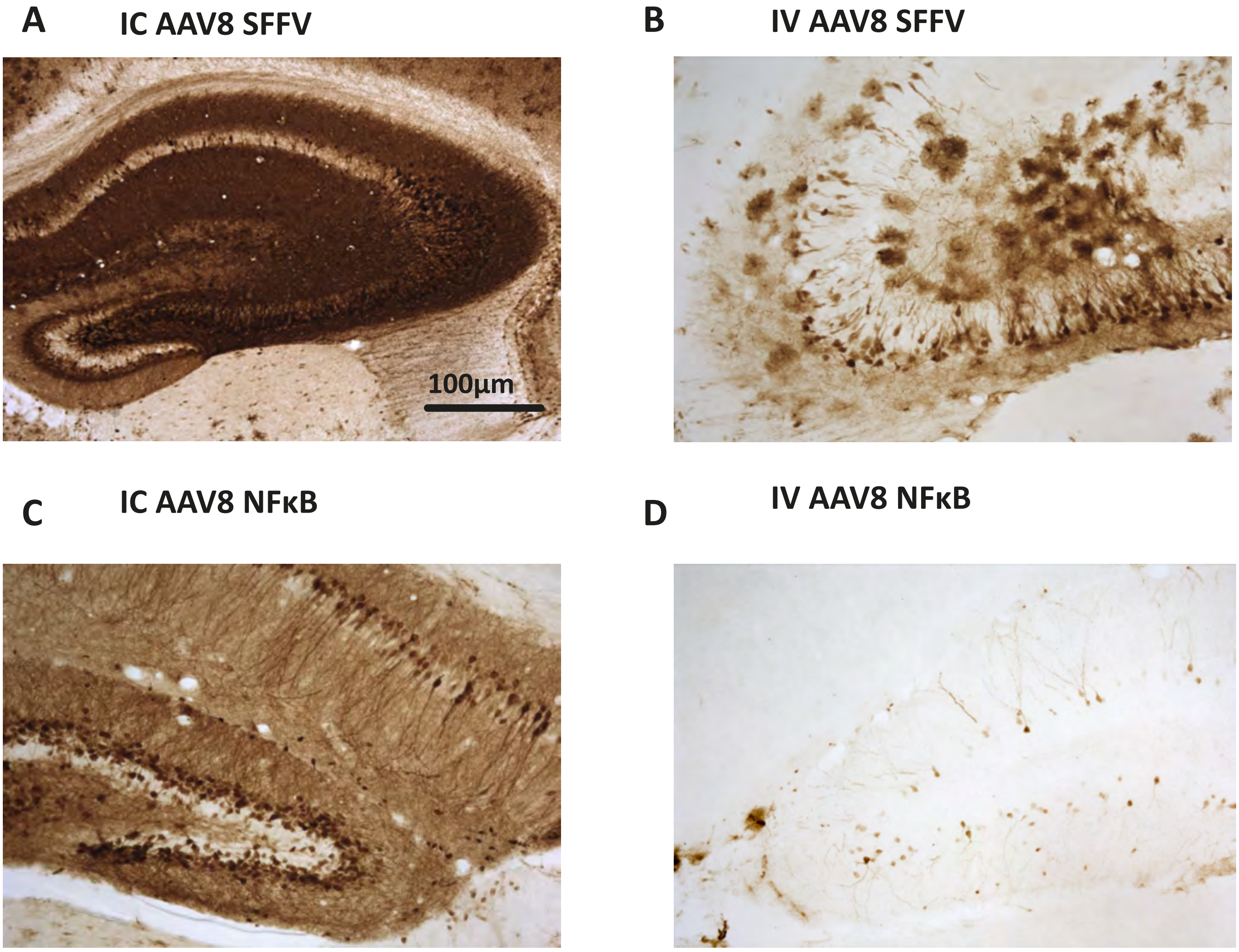

Supplementary figure 7– Neuronal and astrocytic targeting after intravenous or intracranial administration of AAV8 SFFV or NFκB biosensors. Brain sections from injected mice were used to determine GFP positive cells. IC AAV8 SFFV revealed a predominant neuronal transduction within the dentate gyrus and the CA1 and CA3 regions of the hippocampus (A). IV AAV8 SFFV sections showed a mixture of astrocytic and neuronal cell morphology (B). IC AAV8 NFκB targeted neuronal cells (C) whereas IV AAV8 NFκB transduced astrocytes and neurons respectively (D Scale bar 100µm

Supplementary Figure 8

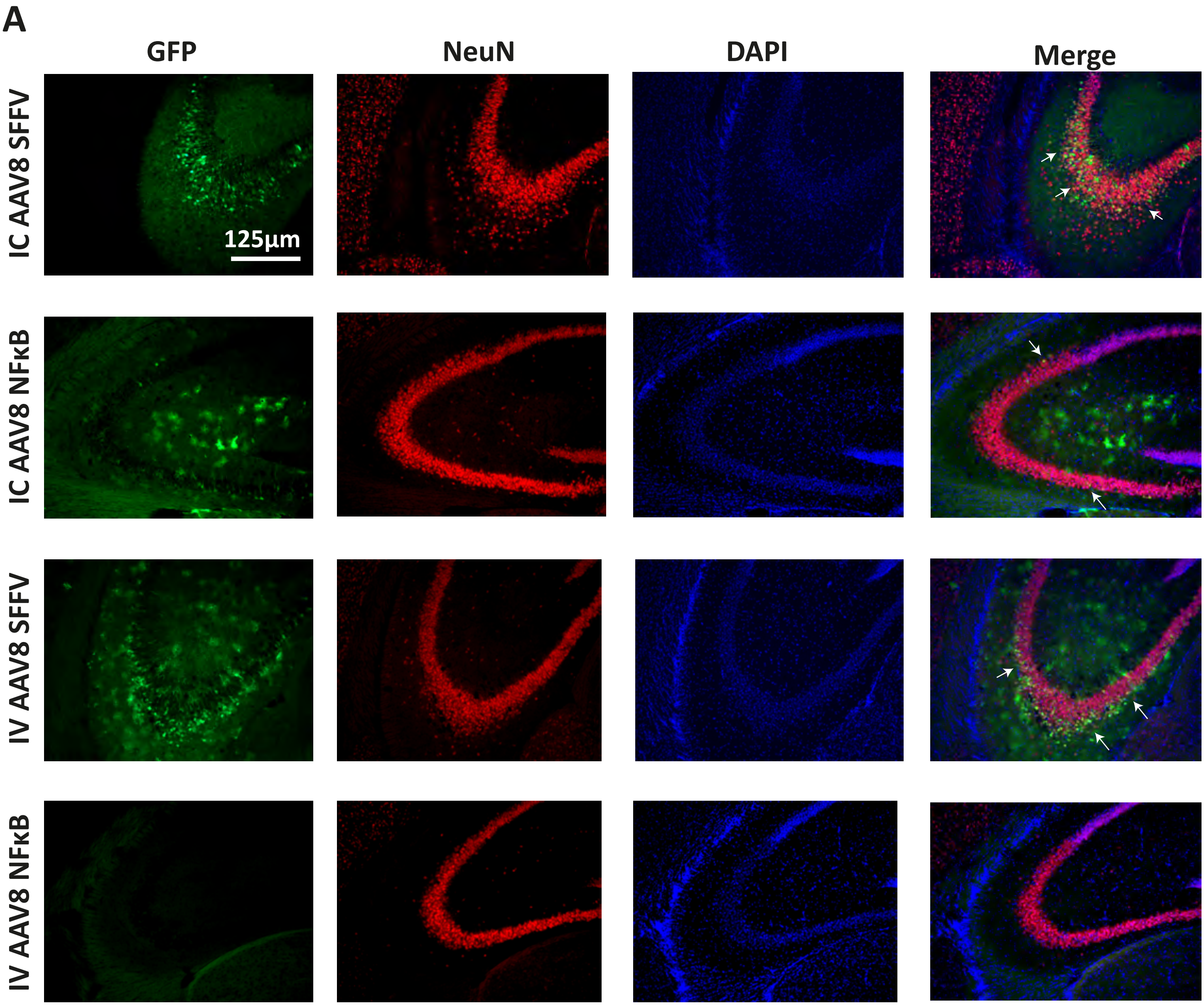

B

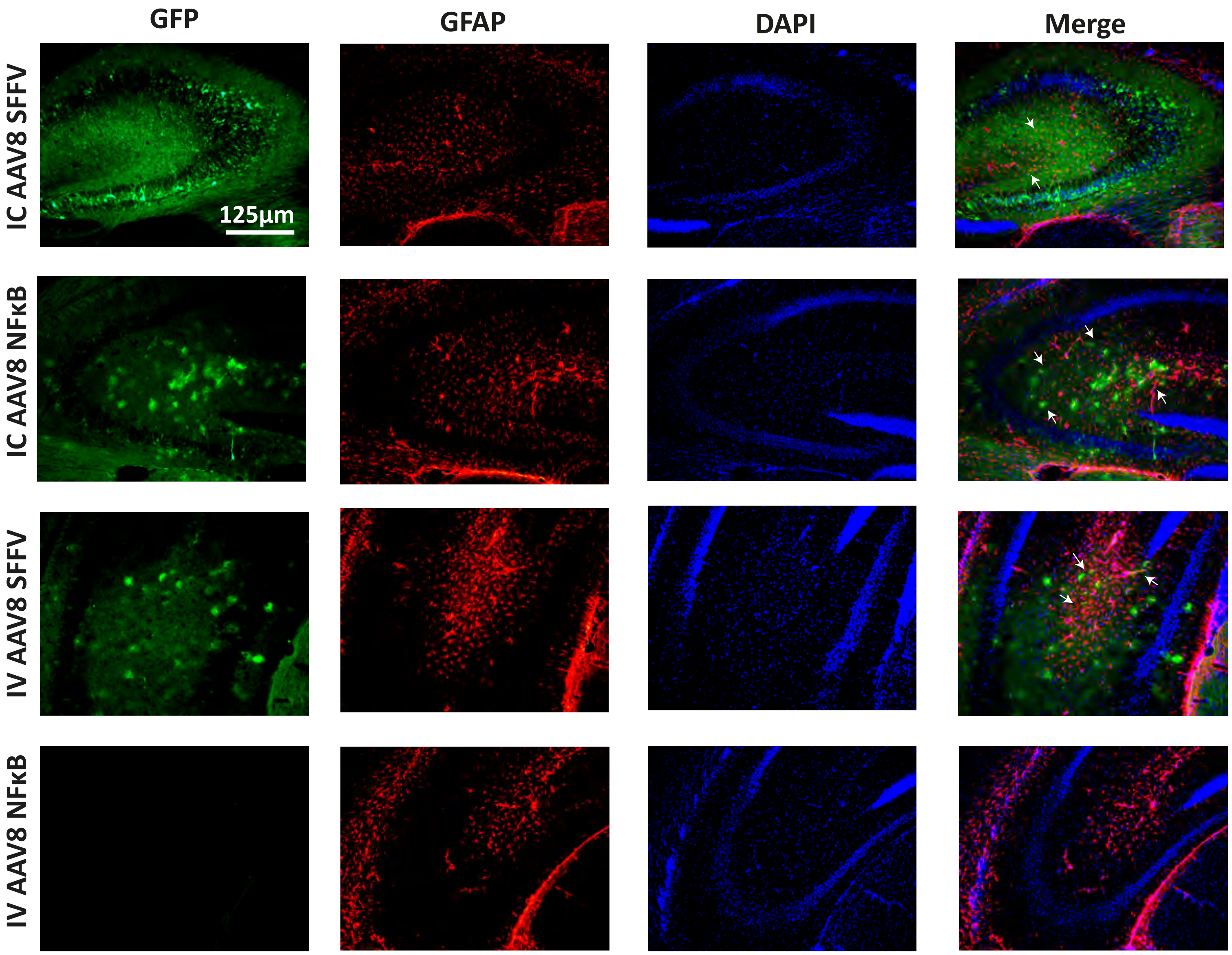

Supplementary figure 8 – Immunofluroecence co-staining to determine neuronal or astrocytic targeting by AAV8 biosensors. Brain sections from injected mice were used to determine neuronal or astrocytic targeting, by co-staining GFP with either NeuN (neuronal marker) and GFAP (astrocytic marker). IC AAV8 SFFV revealed a predominant neuronal transduction within the CA1 and CA3 regions of the hippocampus, indicated by the white arrows in the merge panal. Some neuonal co-staining was observed in the IC AAV8 NFκB and IV AAV8 SFFV brain sections. No GFP positive cells were obsereved in the IV AAV8 NFκB (A). The IC AAV8 SFFV, IC AAV8 NFκB and IV AAV8 SFFV brain sections showed a few cells which co-loaclised with GFAP, indicated by the white arrow (B). Scale bar 125µm.

Supplementary Figure 9

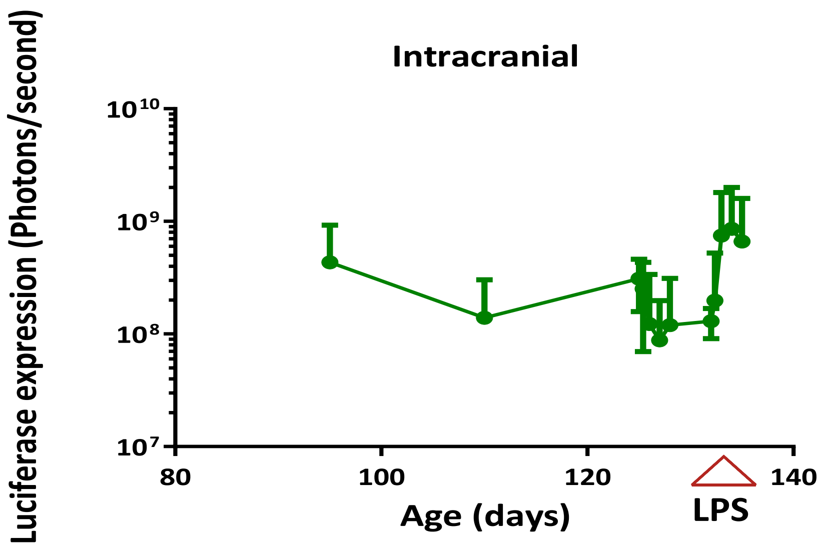

Supplementary figure 9 – Up-regulation of luciferase signal mediated by a single dose of LPS in intracranially injected mice. Bioluminescence imaging persisted more than 4 months over development in mice which received an intracranial administration of AAV8 NFκB biosensor (n=7). At day 132 of development all the mice received a single dose of LPS which resulted in a significant up-regulation in luciferase expression in IC injected mice, p = 0.006 (mean

Supplementary Figure 10

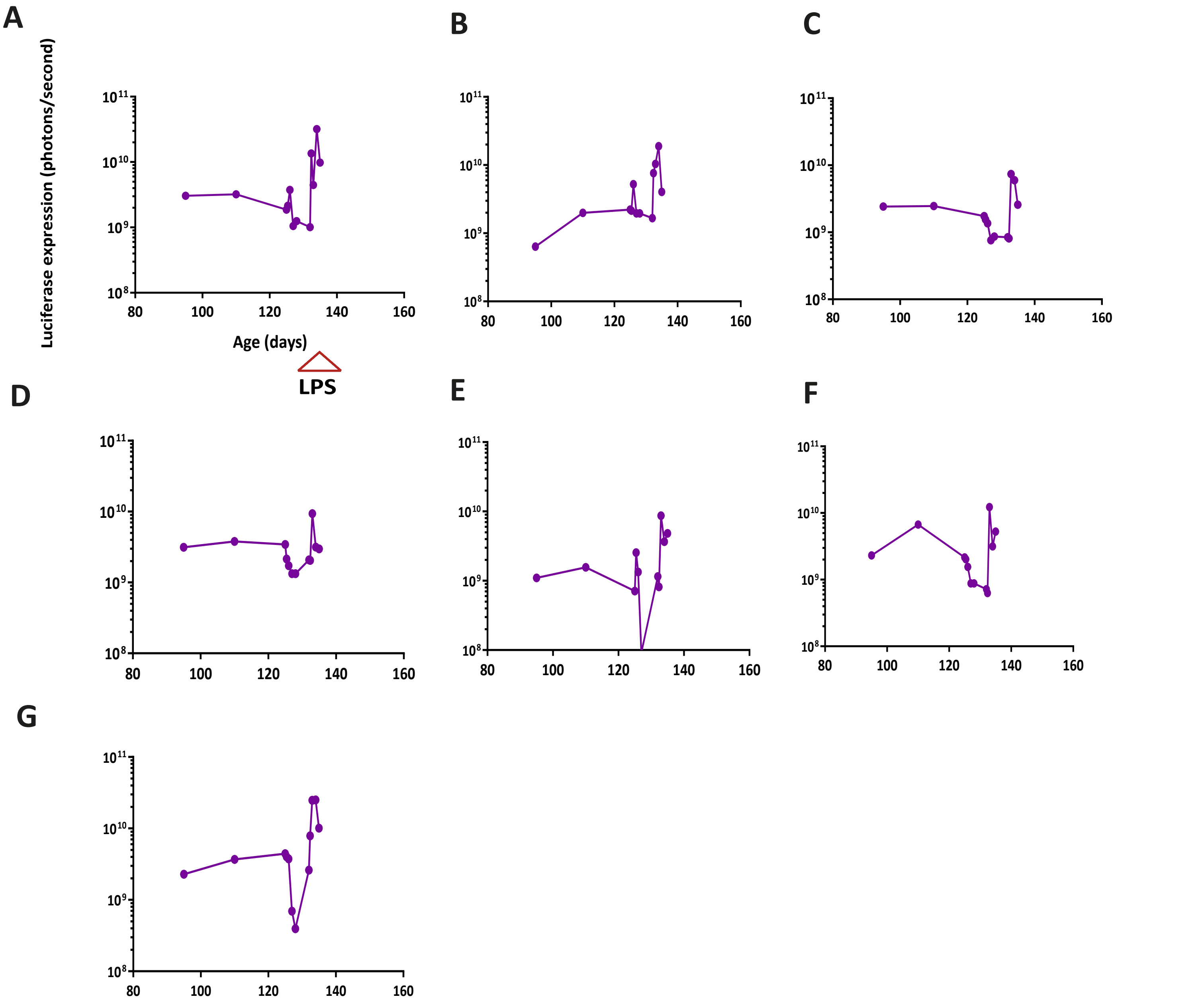

Supplementary figure 10 – Up-regulation of luciferase signal facilitated by a single dose of LPS. 132 post neonatal intravenous injection of AAV8 NFκB biosensor, each mouse received a single injection of LPS. Each mouse responded to LPS and this is shown by the increase in luciferase expression (A-G).

Supplementary Figure 11

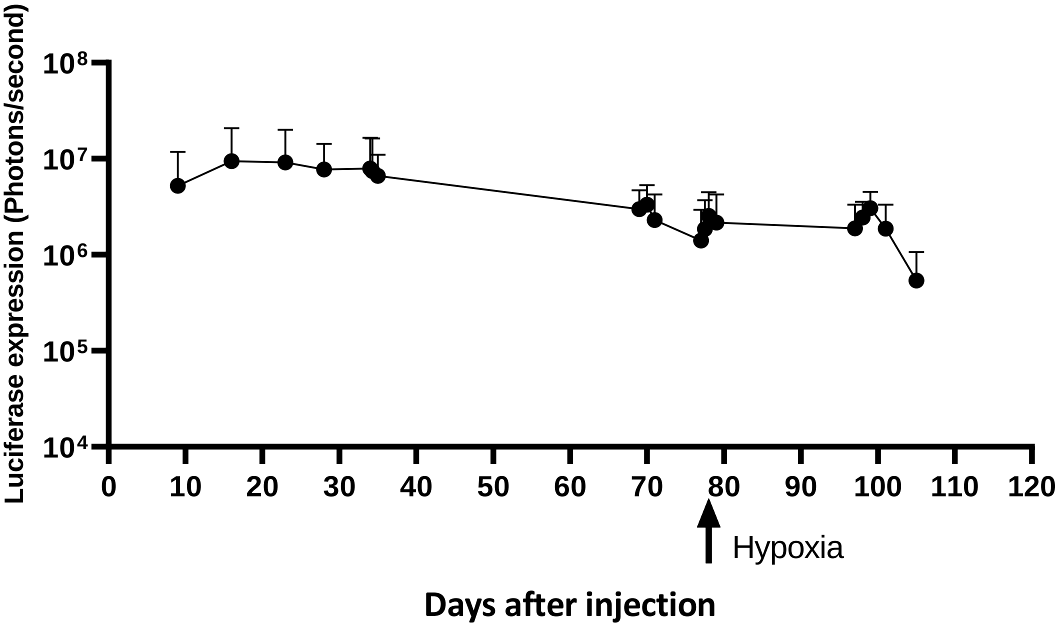

Supplementary figure 11 - Total luciferase expression after hypoxia, in adult mice injected with AAV8 HRE biosensor. Adult CD1 mice (8 week old), received an tail vein injection of $1x10^{10}$ vg/ml (n=10) of the AAV8 HRE biosensor. Luciferase expression was quantified post injection and a burst of imaging was taken before and after hypoxia. The mice were exposed to brief hypoxia (10% oxygen, for 2 hours) at day 77 post inkection and we observed no difference in luciferase expression post-hypoxic exposure.
